# Semi-Elemental and Elemental Formulas for Enteral Nutrition in Infants and Children with Medical Complexity—Thinking about Cow’s Milk Allergy and Beyond

**DOI:** 10.3390/nu13124230

**Published:** 2021-11-25

**Authors:** Elvira Verduci, Silvia Salvatore, Ilia Bresesti, Elisabetta Di Profio, Erica Pendezza, Alessandra Bosetti, Massimo Agosti, Gian Vincenzo Zuccotti, Enza D’Auria

**Affiliations:** 1 Department of Health Sciences, University of Milan, 20146 Milan, Italy; 2 Department of Pediatrics, Vittore Buzzi Children’s Hospital, 20154 Milan, Italy; elisabetta.diprofio@unimi.it (E.D.P.); erica.pendezza@unimi.it (E.P.); Alessandra.bosetti@asst-fbf-sacco.it (A.B.); gianvincenzo.zuccotti@unimi.it (G.V.Z.); enza.dauria@unimi.it (E.D.); 3 Department of Medicine and Surgery, Pediatric and Neonatology Units, Hospital “F. Del Ponte”, University of Insubria, 21100 Varese, Italy; silvia.salvatore@uninsubria.it (S.S.); ilia.bresesti@uninsubria.it (I.B.); massimo.agosti@uninsubria.it (M.A.); 4 Department of Animal Sciences for Health, Animal Production and Food Safety, University of Milan, 20133 Milan, Italy; 5 Department of Biomedical and Clinical Sciences “L. Sacco”, University of Milan, 20157 Milan, Italy; 6 Pediatric Clinical Research Center Fondazione Romeo ed EnricaInvernizzi, University of Milan, 20157 Milan, Italy

**Keywords:** hydrolyzed formula, enteral nutrition, children with medical complexity, neurological children, preterm infants, cow’s milk allergy

## Abstract

Children with medical complexities, such as multi-system disorders and/or neurological impairments, often experience feeding difficulties and need enteral nutrition. They frequently have impaired motility and digestive–absorbing functions related to their underlying condition. If a cow’s milk allergy (CMA) occurs as a comorbidity, it is often misdiagnosed, due to the symptoms’ overlap. Many of the commercialized mixtures intended for enteral nutrition are composed of partially hydrolyzed cow’s milk proteins, which are not suitable for the treatment of CMA; thus, the exclusion of a concomitant CMA is mandatory in these patients for obtaining symptoms relief. In this review, we focus on the use of elemental and semi-elemental formulas in children with neurological diseases and in preterm infants as clinical “models” of medical complexity. In children with neurodisabilities, when gastrointestinal symptoms persist despite the use of specific enteral formula, or in cases of respiratory and/or dermatological symptoms, CMA should always be considered. If diagnosis is confirmed, only an extensively hydrolyzed or amino-acid based formula, or, as an alternative, extensively hydrolyzed nutritionally adequate formulas derived from rice or soy, should be used. Currently, enteral formulas tailored to the specific needs of preterm infants and children with neurological impairment presenting concomitant CMA have not been marketed yet. For the proper monitoring of the health status of patients with medical complexity, multidisciplinary evaluation and involvement of the nutritional team should be promoted.

## 1. Introduction

Enteral nutrition is indicated in patients with an at least partially functioning digestive tract when oral intake is inadequate to meet patients’ needs [1]. The European Society of Pediatric Gastroenterology and Hepatology of Nutrition (ESPGHAN) Committee on Nutrition refers to enteral nutrition supports (ENS) as both the delivery of nutrients via feeding tubes and the provision of specialized oral nutritional supplements [1]. Enteral nutrition (EN) should be preferred to parenteral nutrition (PN) whenever possible, not only for a better safety profile and lower costs, but mainly for the preservation and stimulation of gastrointestinal functions [1,2,3]. Nonetheless, the crosstalk between gut epithelium, immune system and microbiome remains crucial during critical illness [2,3]. Particularly during infancy, as the gut barrier function develops and the gut–immune system axis is set, enteral nutrition is preferable [3].

Among all clinical situations requiring enteral nutrition, there are children with medical complexity (CMC). CMC may have a multi-system disorder (both congenital or acquired) and/or a severe neurological disease causing functional impairment, which frequently requires hospital- and/or community-based service and dependence on technology and/or multiple treatments for everyday activities [4,5].

CMC often includes impaired motility and digestive–absorbing functions, which may benefit from an elemental or semi-elemental diet. The formulas contain partially or totally hydrolyzed macronutrients, such as fructose or digested fractions of cornstarch and maltodextrin, fatty acid esters or MCT, and amino acids or small peptides as sources of carbohydrates, fats and proteins, respectively [6,7,8]. The choice of elemental or semi-elemental products in complex and critically ill children should be accurately tailored to the individual patient’s needs. Cow’s milk allergy (CMA) should also be considered in some individuals with gastrointestinal, respiratory and/or dermatological manifestations. 

CMA is the leading cause of food allergy in infants and young children, with a prevalence of 2–3% depending on the region and diagnostic criteria [9,10,11,12]. The current standard of care for CMA is based on the strict dietary avoidance of cow’s milk protein-containing foods [12]. Three main categories of formulas are available for CMA treatment: extensively hydrolyzed formulas (eHFs), based on caseins or whey proteins, amino acid-based formulas (AAFs) and non-cow’s milk based formulas, based instead on soy proteins (SFs) or rice peptides (eHRFs) [13]. Most available eHFs contain peptides with a molecular weight <3 kDa [14]. The American Academy of Pediatrics defines a formula as hypoallergenic in case of 90% clinical tolerance (with 95% confidence limits) in infants with proven CMA [15]. To target this outcome of efficacy and safety, the European Commission has limited the amount of immunoreactive protein to be less than 1% of the total content of N-containing substances [16].

In the present narrative review, we specifically focus on the use of elemental and semi-elemental enteral formulas in preterm neonates and in patients with neurological impairments as clinical “models” of medical complexity. We do not address enteral feeding for other conditions such as inflammatory bowel disease and specific metabolic disorders (Table 1), which can benefit from semielemental and elemental formulas in selected cases, which are beyond the scope of the present review. 

## 2. Methods

We performed a narrative review of the literature from the past 15 years. We identified the most relevant published studies, including original papers, metanalyses, clinical trials, and reviews. Case reports or series and letters were excluded. Papers published were searched with the following keywords (alone or in combination): neurologically impaired children, disability children, neurological children, delayed gastric emptying, gastroesophageal reflux disease, constipation and diarrhea, cow’s milk allergy, elemental formula, hydrolyzed formulas, preterm infants, necrotizing enterocolitis, food protein-induced enterocolitis. The following electronic databases were searched: PubMed, Scopus, EMBASE and Web of Science. As Supplemental Materials we provide a flow chart for the eligibility criteria of the included studies. 

## 3. Food for Special Medical Purposes and Nutritional Aspects: General Considerations

In accordance with Commission Delegated Regulation (EU) 2016/127), enteral nutrition products are classified as medical foods, known as enteral feed if this is the sole source of nutrition or supplemental feed when used in addition to normal food. In infancy the nutritional composition of infant formulae and follow-on formulae should be considered [17]. As reported by the ESPGHAN Committee on Nutrition, enteral nutrition is mainly offered as liquid ready-to-feed formulas with variable composition in terms of energy, macronutrients and other selected nutrients, which should be age- and disease-adapted. When enteral feeding is indicated, low-energy formulae (<1 kcal/mL) are much less used than formulae with energy density of 1 kcal/mL, while in subjects with increased energy requirements, higher-energy density formulas (1.5–2 kcal/mL) are recommended [1]. In case of normal motility and intestinal function, the preferred option is usually a standard polymeric formula, which is designed to mimic normal diet by providing carbohydrates (such as corn syrup and maltodextrin), intact proteins (mainly from cow’s milk) and fat (such as canola, palm and/or safflower oil) in nonhydrolyzed forms [7]. When impairment of intestinal function is present, low-molecular formulas, which contain oligopeptides derived from hydrolyzed protein or free amino acids, are indicated. 

Moreover, some nutritional enrichments of formulas has proven beneficial in improving the gastrointestinal health of critically ill children, including fiber [18,19], prebiotics/probiotics [20], medium-chain triglyceride (MCT) [21,22] and omega-3 [23]. Finally, enteral formulas are usually gluten- and mostly lactose-free [1,24]. They can be classified as reported in Table 1.

The extent of hydrolysis is a key point to take into account for the choice of the formula in children with a cow’s milk allergy, but also with other enteropathy and motility disorders. Semi-elemental formulas include not only eHF but also partially hydrolyzed formulas (pHFs). The difference between eHFs and pHFs is mostly established by the molecular weight profile. Both eHFs and pHFs contain a wide range of peptides, but in pHFs the vast majority of the peptides are <5 kDa (with a size distribution of 3–10 kDa), while in eHFs almost all peptides have a molecular weight <3 kDa [14]. Considering that peptides ≥3 kDa are able to elicit an allergic reaction, it is clear that pHFs are not intended for infants with CMA, and that a reaction to eHFs is still possible in a very small proportion (5%) of cow’s milk-allergic subjects [14]. Although they may have some use in milder forms of digestive disorders [25], which are a frequent complaint in these patients, partially hydrolyzed formulas should not be use for treatment of suspected or proven CMA [12].

Elemental formulas include amino acid-based formulas (AAFs). While these formulas are the only truly hypoallergenic ones and represent the first nutritional choice for severe CMA, the presence of free amino acids increases the osmolarity of the formula that may be related to persistent symptoms in a critically ill child, especially if malabsorption is present [26].

## 4. Preterm Infants

**Figure 1:**
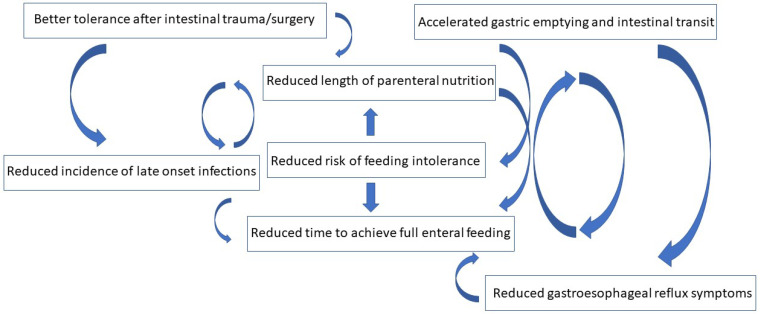
Potential benefits of the use of hydrolyzed formulas in preterm infants.

Although human breast milk is currently recommended as the first choice to feed preterm infants [27], the use of hydrolyzed cow’s milk formula (HMF) as a sole or supplemental feed source in this population has increased since the late 1990s. The perceived benefit of this type of milk is mainly the reduced risk of feeding intolerance, which might subsequently reduce the risks related to prolonged parenteral nutrition (see Figure 1). However, the HMF is not free from adverse effects. Because of the process transforming proteins into smaller peptides, this type of milk has increased osmolarity, and potentially less bioavailability and bioactivity of micronutrients and immunoglobulins [28,29]. Concerns have also arisen about the association with chronic inflammatory metabolic and neurogenerative diseases later in life [30]. According to a recent Cochrane review, the evidence available so far is not robust enough to draw a broad conclusion regarding the short- and long-term effects of hydrolyzed milk compared to the standard formula in preterm infants [31]. When human milk is not available, HMF might be considered as a starter formula or rescue treatment in babies with CMA, feeding intolerance and/or gastroesophageal reflux symptoms. In fact, the HMFs are commonly associated with accelerated gastric emptying and intestinal transit, more effective enteric peptide digestion, and stimulation of small intestinal enzymatic and motilin activity [29,32]. According to one report, time to achieve full enteral feeding was reduced in a study in very preterm infants [32], but no statistically significant difference has been found between the hydrolyzed and the standard formula in other trials [29,31,33,34]. A recent study compared the effects of two formulas, intact protein formula (IPF) and extensively hydrolyzed protein formula (eHF), on gastric emptying in preterm infants. Real-Time Ultrasonoghaphy found that median gastric emptying time at day 14 was significantly faster for the eHF compared with the IPF group. However, it did not predict feeding tolerance [35].

Moreover, even if HMFs are well tolerated by preterm infants and harmful effects have not been reported so far, several studies have demonstrated that their nutritional value is lower than that of intact protein formulas. Weight gain was reported to be slower among infants fed by HMF, due to a lower nitrogen/protein absorption rate [34,36]. Conversely, no differences were found regarding length gain and head circumference growth [34,36]. These findings suggest that the HMFs have lower nutritional efficiency, and an average increase of 10% in protein content is desirable to achieve the same protein retention rate as standard preterm formula [33,37]. Bone mineralization seemed not to be affected by the type of milk, although a lower mineral absorption was often experienced by neonates fed with HMF [37]. No data are currently available in regard to the influence of HMFs on trace elements and vitamin absorption. It is noteworthy that hydrolyzed milk formulas have been markedly improved in the last few years, through the selection of the protein source (formulas containing various whey:casein ratios and acidic whey instead of sweet whey), an increased protein:energy ratio, the addition of specific amino acids and the enhancement of hydrolysis technologies. Several improvements have also been put in place to reduce mineral retention, which involve increasing calcium and phosphorus content and selecting high-bioavailability minerals. How the use of hydrolyzed formula milk influences necrotizing enterocolitis (NEC) onset is still debated. A meta-analysis including data from five trials showed no differences compared to formula-fed babies, although there were several limitations in the studies analyzed [31]. Although it is a quite common perception among neonatologists that milk with hydrolyzed proteins might be better tolerated in compromised infants or neonates with intestinal trauma, there is insufficient evidence supporting the use of HMF in the regime of feeding after NEC or gastrointestinal surgery.

A separate discussion regards the management of food protein-induced enterocolitis (FPIES) in preterm newborns or infants. FPIES is a non-IgE-mediated food allergy, and cow’s milk is the most determinative trigger food [38]. In the neonatal period, acute FPIES may mimic countless different conditions, ranging from chirurgical conditions to infectious diseases and inborn errors of metabolism [39]. In preterm newborns, acute FPIES should be carefully differentiated, in particular from necrotizing enterocolitis [40]. In neonates, both conditions may present with feeding difficulties, abdominal distension, vomiting, diarrhea, and systemic findings such as lethargy, apnea and hypotension. Despite the similar clinical findings, the management of these two conditions is remarkably different. While NEC requires pharmacological treatments with antibiotics and in most severe cases chirurgical intervention, FPIES may be successfully managed via dietary management, by avoiding cow’s milk proteins [41].

In babies fed with human milk, the exclusion of cow’s milk proteins from the maternal diet often resolves symptoms; in newborns or infants not fed with human milk, a hydrolyzed formula should be introduced. While DRACMA Guidelines and BSACI Guidelines recommend the use of an elemental formula [12,42], International Guidelines [41], NIAID GuideLines [43], and ESPGHAN Guidelines [9] recommend an extensively hydrolyzed formula as the first nutritional choice, and only in cases of symptom persistence or growth failure should they be given an elemental formula. It is noteworthy that the existing guidelines made general recommendations, not specifically referring to premature newborns. According to our experience and the few reports in the literature regarding FPIES in preterms, patients who failed to recover with an extensively hydrolyzed formula all responded to the amino acid formula [40]. For this reason, an elemental diet based on an amino acidic formula might be considered as the first-line approach in these fragile populations of preterm newborns, due to the common immature absorption functions in these subjects [40].

Uncertainty characterizes the incidence of late-onset infections and of allergic diseases (atopic dermatitis, gastrointestinal symptoms, wheezing) after the first year of life [31]. Then, as for term infants, the familiarity appears to be the main risk factor for developing allergic diseases [37]. Last, there is a lack of evidence regarding hospital stay duration, mortality and neurodevelopmental outcomes [31]. In summary, although potentially promising, the clinical benefits of HMFs in preterm infants have not been demonstrated yet, either from a short- or a long-term perspective. Thus, no consensus is currently available to tailor the use of hydrolyzed milk in preterm infants.

Further high-quality studies with large number of infants enrolled are thus urgently needed to clarify these aspects.

## 5. Neurological Children

### 5.1. Cow’s Milk Allergy (CMA) in Neurological Children

CMA may occur in children with critical illness and neurological impairment, but its prevalence is uncertain. Because CMA may be immunoglobulin E (IgE)- or non-IgE-mediated and its clinical manifestations are highly variable in type and severity, with symptoms overlapping many other conditions, its recognition is often challenging [12,44]. The common diagnostic approach of CMA includes testing for specific IgE or oral challenge. Nonetheless, a diagnostic elimination diet is also considered for children with negative IgE [9,12]. The current standard of care for CMA is based on the strict dietary avoidance of cow’s milk protein-containing foods.

To our knowledge, epidemiological data reporting CMA in pediatric neurological patients are scarce. A cohort study was conducted in 2012 in Centro de Rehabilitación Infantil Teletón de Puebla [45], enrolling 145 children, aged 6 months to 5 years, with different disabilities (Cerebral palsy, Cerebral dysgenesis, Genetic syndrome, Birth asphyxia, Down’s syndrome, Axonal brain injury). For the identification of probable cases of CMA, an exploratory questionnaire of related clinical manifestations was applied.

Twenty-six cases of CMA (corresponding to 18% of children) were detected. The most frequently reported manifestations were from the respiratory tract, followed by digestive, neurological and dermatological symptoms. The gastrointestinal findings included constipation (in 20/26 children), colic-like abdominal pain (in 20/26), refusal of food (in 20/26) and regurgitations (in 19/26 subjects). Most of the children with constipation had been treated with laxatives without response. Children with clinical data of CMA were started on a cow’s milk protein exclusion diet for 12 months. After the intervention, all children were clinically reassessed, and the same questionnaire was used. For children with atopic dermatitis, the severity of symptoms was measured with the Score for Atopic Dermatitis (SCORAD), which significantly decreased after the cow’s milk protein elimination diet (*p* = 0.001). Families of all patients reported a significant decrease in the frequency of respiratory symptoms (*p* = 0.001) and, in children receiving pulmonary rehabilitation, in the amount of secretions expelled during bronchial hygiene therapies associated with an improvement in basal oxygen saturation. On-diet constipation persisted in only two patients, and food refusal in one, while abdominal pain and regurgitations subsided in all previously affected patients. Authors concluded that exclusion diet in children with CMA and disabilities should be considered to reduce different persistent symptoms and improve the quality of life. It is noteworthy that in this study, all children did not undergo an oral provocation test after exclusion diet, as recommended by the Guideline, to make a certain diagnosis [9,12,13].

Actually, screening for food allergies is not recommended in all children with neurological disorders. At present, the exact prevalence of CMA based on a proper diagnostic work-up with elimination diet, followed by the oral provocation test, is still unknown.

### 5.2. Delayed Gastric Emptying and Gastroesophageal Reflux Disease (GERD) in Neurological Children

Gastrointestinal and feeding disorders develop more frequently in patients with central nervous system abnormalities [46].

Particularly in children with neurological impairment (NI), gastrointestinal symptoms are frequent, and oral and gut motility are often compromised, causing dysphagia, feeding disturbance, malnutrition, delayed gastric emptying and gastroesophageal reflux (GERD) [47]. Factors leading to GERD include prolonged supine position, increased intrabdominal pressure secondary to spasticity, hiatus hernia, scoliosis, seizures, and esophageal and antroduodenal dysmotility [46]. The nutrient and energy losses are often due to frequent regurgitation and vomiting, which aggravate malnutrition [48]. 

The type of nutrition and formula choice for these children should consider the individual energy requirements determined by the neurological condition and weight gain, the oropharyngeal function, the swallowing capacity and the gastrointestinal motility. Two reports showed a reduction in reflux episodes and reflux duration in children fed with whey protein-based formulas with a high pectin content [49,50].

The animal model highlighted that oral casein intake was associated with delayed gastric emptying, while whole whey proteins had a slower intestinal transit [51]. 

A recent systematic review [52] on the impact of protein type and degree of hydrolysis on gastric emptying included eight studies comparing breast milk versus whole whey/casein formulas, whole protein casein versus whey feeds, whole protein versus hydrolyzed protein, and hydrolyzed casein versus hydrolyzed whey. Breast milk resulted in faster gastric emptying than formula in all studies. A comparison of healthy children and children with cerebral palsy showed differences in gastric emptying. In children with cerebral palsy and GERD, the formula containing whole casein proteins resulted in slower gastric emptying than the formula with predominantly whey proteins. No significant difference was found between extensive or partially hydrolyzed formula in terms of GERD symptoms. In conclusion, although it seems that whey protein-based formulas allow a faster gastric emptying, different methodologies, formula compositions and patient groups make it difficult to draw firm conclusions. 

Table 2 summarizes the studies [53,54,55,56] assessing gastric emptying after feeding with formulas with different types of proteins and hydrolysis. The patients included in these studies had cerebral palsy or psychomotor retardation, and had gastrostomy and/or fundoplication. The octanoic acid breath test and/or a validated symptoms questionnaire were used to assess improvement in GI symptoms.

### 5.3. Constipation and Diarrhea in Neurological Children

Neurological patients frequently suffer from constipation because of non-ambulation, intestinal dysmotility, hypotonia, and typically reduced fluid and fiber intake [47]. The addition of fiber to enteral formulas has long been considered to accelerate gut transit time, suggesting that fiber could be advantageous in enterally fed individuals suffering from slow-transit constipation [57]. Nevertheless, a systematic review and meta-analysis [18] analyzing the clinical and physiological effects of fiber-enriched formulas found that the use of a single type of fiber (soluble or insoluble) can lead to the opposite effects, including increased gas production in the case of highly fermentable and soluble fibers such as guar gum or inulin, or delayed transit in the case of insoluble fibers such as cellulose. Hence, the fiber content of an enteral formula should ideally mirror the balanced and different fibers consumed naturally in a normal diet [18]. It is noteworthy that the content of dextrose equivalents (DEs), i.e., monosaccharides, disaccharides and trisaccharides, which are highly fermentable, can increase gastrointestinal symptoms such as distension, bloating and diarrhea [58]. Furthermore, although there are still conflicting and incomplete data, MCT addition to enteral formulas can be beneficial because this type of lipids can be easily absorbed, and can ameliorate symptoms such as chronic diarrhea, impaired growth, and poor appetite. Moreover, they can improve the digestion and absorption of micronutrients such as minerals and vitamins, reducing the rate of anemia and other vitamin and mineral deficiencies [59]. It seems that they may improve duodena–ileal transit, without the production of cholecystokinin [60], and in intensive care adult patients the administration of MCT 20% energy + carnitine + taurine via the enteral route has been shown to reduce intolerance, defined as diarrhea or vomiting (2.8–8.2%), gastric stagnation (4.2–8.2%) or abdominal distension (26.8–43.8%) [61].

We present below two cases (see Box 1 and Box 2) of children with a complex clinical situation, which highlights the challenge of the nutritional management of critically ill children.

Box 1 **Clinical Case 1.**

The first case relates to a 2-year-old girl suffering from epileptic encephalopathy, congenital microcephaly, cortical blindness and severe psychomotor retardation. At 5 months of age, immediately after ingestion of the formula, she experienced vomiting, angioedema of the auricle and lips and hives/urticaria on the face and neck. S-IgE (milk 2.7 kUa/L, casein 2.14 kUa/L, alfalactalbumin 0.30 kUa/L, betalactoglobulin 10.7 kUa/L) and prick tests were performed, and CMA was diagnosed. She started an elimination diet with aminoacidic formula, later continued with complementary feeding excluding cow’s milk proteins.

At 10 months of age, due to significant feeding difficulties with dysphagia, sialorrhea, vomiting and weight loss, a nasogastric feeding tube and subsequent percutaneous endoscopic gastrostomy (PEG) were employed. She continued enteral nutrition via PEG, meeting 100% of total daily energy requirement, with aminoacidic formula.

Due to poor control of seizures, genetic analysis was performed and diagnosis of GLUT1 deficiency syndrome was made. A ketogenic diet was therefore started with eHF plus MCT oil. However, this approach did not allow an adequate intake of nutrients to meet the child’s requirements, and was difficult for her family to manage.

At 3 years of age, the child was hospitalized for multi-day seizures, with transient hypertonia of the limbs, fixed gaze, head deviation and myoclonias of the limbs. On the basis of the clinical course, the neurologist prescribed an increase in the ketogenic ratio of the diet, which required the use of a formula specifically intended for the nutritional treatment of drug-resistant epilepsy, containing cow’s milk proteins. A food challenge test was therefore performed via PEG, in the hospital setting, with the administration of increasing doses of specifical ketogenic formula, initially in bolus and then via nutritional pump. The procedure was well tolerated, and the test proved negative: therefore, the child was able to start the ketogenic diet with a 4:1 ratio via PEG, which led to clinical improvement and growth curve normalization.

Box 2 **Clinical Case 2.**

Another interesting case concerns a 13-year-old boy with x-linked adrenoleukodystrophy with symptomatic epilepsy, spastic tetrapareris, dysphagia and adrenal insufficiency.

At 7 years of age, due to feeding difficulties, PEG was positioned and he started feeding with a semi-elemental, normocaloric (1 kcal/mL), nutritionally complete formula. The formula was well tolerated, without any symptoms except for chronic constipation.

At 12 years of age, the boy was admitted to hospital for PEG-J positioning, and gradual resumption of enteral nutrition was started with a normocaloric (1 kcal/mL) formula containing soluble fibers, to decrease constipation. On the label, the formula reported “composed of small peptides” with no contraindications for CMA. However, after the first administration, the child experienced an anaphylactic reaction which required adrenalin injection. Tests for specific food IgE were performed and were positive for milk (15.60 ku/L) and casein (18.30 ku/L) confirming the diagnosis of CMA. Consequently, an elemental formula based on free amino acids was started with symptom resolution and patient well-being.

The present cases address the need for investigating the presence of a concomitant food allergy in neurological patients, and more generally in patients with medical complexity, in cases of severe and persistent gastrointestinal symptoms and/or intolerance to the formula in use. The importance of verifying the tolerance before starting a specific dietary treatment (e.g., ketogenic diet) for severe and intractable seizures has also been shown.

## 6. Conclusions

This review highlights how challenging the nutritional management of critically ill children can be. CMA can occur in CMC, such as those with neurological impairments, or in preterm children.

Since these children often experience impaired motility and digestive–absorbing functions related to their underlying condition, the risk of misdiagnosis is high; when gastrointestinal, respiratory, or dermatological symptoms occur, CMA should always be considered.

In preterm children with gastrointestinal symptoms, when breastfeeding is not possible, HMF might be considered as a starter formula or rescue treatment. Nevertheless, consensus is still lacking regarding the best nutritional approach in preterm newborns, and the clinical benefit of HMF still needs to be further clarified.

When a preterm infant is suffering from FPIES, an elemental diet, with a free amino acid formula, could be used as a first choice.

The currently available data regarding the treatment of CMA in children with neuro-disabilities are scarce. In Table 3, we summarize the pros and cons of elemental and semi-elemental formulas for both neurologic and preterm children.

Even though many of the commercialized formulas for enteral nutrition are composed of hydrolyzed cow’s milk proteins, the extent of hydrolysis is often not clearly specified, making them not suitable for CMA treatment. As stated by current guidelines, in the case of CMA, only an extensively hydrolyzed or amino-acid based formula, or, as an alternative, extensively hydrolyzed formulas derived from rice or soy with nutritional adequacy and demonstrated efficacy, must be used.

Currently, enteral formulas for CMA include extensively hydrolyzed formulas and amino acid formulas intended for infants and young children; hypoallergenic formulas, specifically tailored to the specific needs of preterm infants and children with neurological impairment and concomitant CMA, are lacking 

For instance, elemental formulas with high energy density (1.5 kcal/mL) may help to fill the energy requirements that increase with growth, which is particularly important if the formula is used as the only source of feeding. In addition, amino acidic formulas with added fiber may be useful in cases of constipation due to underlying diseases. Finally, the labels of enteral nutrition formulas should clearly indicate the source and degree of the hydrolysis of proteins, and should include a clear contraindication for patients with CMA.

In conclusion, in critically ill children, a multidisciplinary approach, and the use of a nutritional team including a pediatrician, a dietician, a speech therapist, an ENT specialist, an allergist and a gastroenterologist, should be promoted to improve the management of children with medical complexity.

## Figures and Tables

**Table 1:** Different types of classification for enteral formulas.

Energy Density Classification		
Low	Normal	High
<1 kcal/mL	1 kcal/mL	>1 kcal/mL (1.5–2 kcal/mL)
**Molecular content of different formulas**		
Polymeric	Oligomeric or semi-elemental	Monomeric or Elemental
Cow’s milk/soy protein Polysaccharides Triglycerides (LCT) Good palatability Low osmolarity (<300 mOsmol/L) Less expensive	Hydrolyzed protein Maltodextrin + oligosaccharides MCT (≥40%) and LCT Reduced palatability Medium osmolarity (300–500 mOsmol/L) More expensive than polymeric one	Free amino acids Oligosaccharides LCT and MCT (35%) Poor palatability Possible high osmolarity (>600 mOsmol/L) Expensive formula
Generally, gluten-free and lactose-free or lactose in low amounts. Usually, a mixture of bulking and fermentable fiber added in selected commercial formulas.		
**Types of formulas and indications**		
Standard	Balanced mix of all the essential nutrients	Subjects with normal motility and gut functions
Disease-specific formulations	Modification of 1 or more nutrients depending on the metabolic condition of the disease	**Inflammatory bowel disease:** - containing immuno-modulating and anti-inflammatory factors - polymeric feeds for oral and tube feeding - sole source of nutrition or supplement - normal ED, balanced (1 kcal/mL, Pr 14%, Lip 42%, Ch 44%)
		**Renal disease:** - reduced protein content (Pr equivalent 1.5 g/100 mL) - reduction in P, Cl, Ca, K, and Vit A
		**Liver disease:** - increase in branched AA (30%), MCT (50%) and Zn - reduction in Na and Cu
		**Cystic fibrosis:** - high ED, MCT and LCPUFA, vitamins
		**Inborn errors of metabolism:** different formulations on the basis of the enzymatic deficit
		**Ketogenic diet for refractory epilepsy:** - high-fat (71–90% energy) and carbohydrate-restricted (5–19% energy) diet that contains adequate amount of protein - range of KD (g fat/g protein + g carbohydrate) ratio used is 3:1 or 4:1 with respect of tolerance, ketosis and side effects - enrichment with LCPUFA (DHA, AA), vitamins and minerals
		**Respiratory failure:** - high lipids (50%E)
**Formula preparation**		
Ready to feed	Liquid concentrate	Powder
No mixing required Decreased risk of contamination due to decreased manipulation Considered commercially sterile Most expensive form Limited shelf life	1:1 ratio concentrates to water Considered commercially sterile Caloric concentration of final product can be manipulated must be used within 24 hours of mixing	Usually, 2 ounces of water to 1 scoop of powder (14 g/100 mL) Not sterile Preparation required for each meal Less expensive

ED: Energy density. Pr: Proteins. Lip: Lipids. Ch: Carbohydrates. AA: Amino acids. MCT: Medium-chain triglycerides. mOsmol: Milliosmole. LCPUFA: Long-chain polyunsaturated fatty acids. KD: Ketogenic diet.

**Table 2:** Studies conducted to evaluate gastric emptying after intake of formulas with different types of protein and hydrolysis. T ½: half emptying time.

Study	Formulas	Study Design, Population and Sample Size	Assessment Tools	Results
Savage 2012 [54]	Formula 1: 82% casein–18% whey protein Formula 2: 50% casein–50 % whey protein Formula 3: 100% partially hydrolyzed whey protein	RCT crossover 13 children with CP, gastrostomy and fundoplicatio.	MII-ph OBT Symptoms	MII-pH Formula 1: at T½ 56.6 min Formula 2: at T½ 33.1 min Formula 3: at T½ 39 min Formula 1 vs. 2/3 (*p* = 0.033) Formula 2 had significantly lower reported symptoms score (* *p* = 0.035) and lower pain score (** *p* = 0.014) vs. Formula 3
Brun 2012 [53]	Formula 1: 100% casein Formula 2: 100% whey protein Formula 3: 100% amino acids Formula 4: 40% casein–60% whey protein	RCT crossover 15 children with CP, gastrostomy	OBT Validated symptoms questionnaire	Formula 1: at T½ 153 min Formula 2: at T½ 82 min Formula 3: at T½ 74.4 min Formula 4: at T½ 63.3 min (*p* < 0.001) and symptoms only at T½ 16 min.
Brun 2013 [56]	Formula 1: 100% casein Formula 2: 40% casein–60% whey protein	RCT 10 children with CP, gastrostomy and Nissen fundoplicatio 10 children with CP and gastrostomy	OBT Validated symptoms questionnaire	Formula 1: at T½ Nissen 110 min vs. no Nissen 181 min Formula 2: at T½ Nissen 50 min vs. no Nissen 85 min Formula 1: Nissen symptoms 3/10 vs. no Nissen symptoms 2/10 Formula 2: Nissen symptoms 7/10 vs. no Nissen symptoms 0/10 (*p* < 0.001)
Minor 2016 [55]	Formula 1: Standard polymeric formula Formula 2: 100% partially hydrolyzed whey protein	Retrospective study 13 children with psychomotor retardation (11 with fundoplicatio)	Clinical evaluation of symptoms	Formula 2: improvement in tolerance in 12/13

RCT: randomized control trial; CP: cerebral palsy; MII-pH: Multichannel Intraluminal Impedance pH testing; T½: half gastric emptying time; OBT: octanoic acid breath test.

**Table 3:** Pros and cons of elemental and semi-elemental formulas both for neurologic and preterm children.

	Elemental Formulas	Semielemental Formulas
Neurologically impaired children	Pros Anallergic Lactose-free Suitable among patients with gastro-intestinal impaired digestion and absorption compared to polymeric formula Cons Risk of high osmolarity and related increased diarrhea Fiber-free Higher cost compared to semi elemental formulas and polymeric formulas	Pros Hypoallergenic based on the hydrolysis degree Improve gastric emptying and related symptoms of GER Mostly lactose-free Formulas with added fiber are available on the market Lower osmolarity compared to elemental formula Suitable among patients with gastro-intestinal impaired digestion and absorption compared to polymeric formula Cons Risk of allergic reactions in 5–10% of children with concomitant CMA Higher cost compared to polymeric formulas
Preterm infants	Very limited data available Pros Anallergic Increased digestion and absorption compared to polymeric formulas Reduced feeding intolerance Cons The content of protein may be inadequate to normal weight gain Risk of high osmolarity and related increased diarrhea Lactose-free Possible altered development of taste Higher cost compared to semielemental formulas and polymeric formulas	Limited data available Pros Hypoallergenic based on the hydrolysis degree Improves gastric emptying and related symptoms of GER Lower osmolarity compared to elemental formula Better digestion and absorption compared to polymeric formula Reduced feeding intolerance and length of parenteral nutrition Reduced time to achieve full enteral feeding Cons The content of protein may be inadequate to normal weight gain Potential risk of reduced bioavailability and bioactivity of micronutrients and immunoglobulins Possible altered development of taste Potential metabolic consequences later in life Risk of allergic reactions in 5–10% of children with concomitant CMA Mostly lactose-free Higher cost compared to polymeric formulas

## Data Availability

Not applicable.

## Supplementary Materials

The following are available online at https://www.mdpi.com/article/10.3390/nu13124230/s1, Figure S1: Flow chart with elegibility criteria for inclusion in this paper.
