# Supplementary figures and images for: Semi-Elemental and Elemental Formulas for Enteral Nutrition in Infants and Children with Medical Complexity—Thinking about Cow’s Milk Allergy and Beyond
Source: Nutrients. 2021 Nov 25;13(12):4230. doi: 10.3390/nu13124230 (PMC8707725; doi:10.3390/nu13124230)

Figure S1. Flow chart with eligibility criteria for inclusion in this paper.

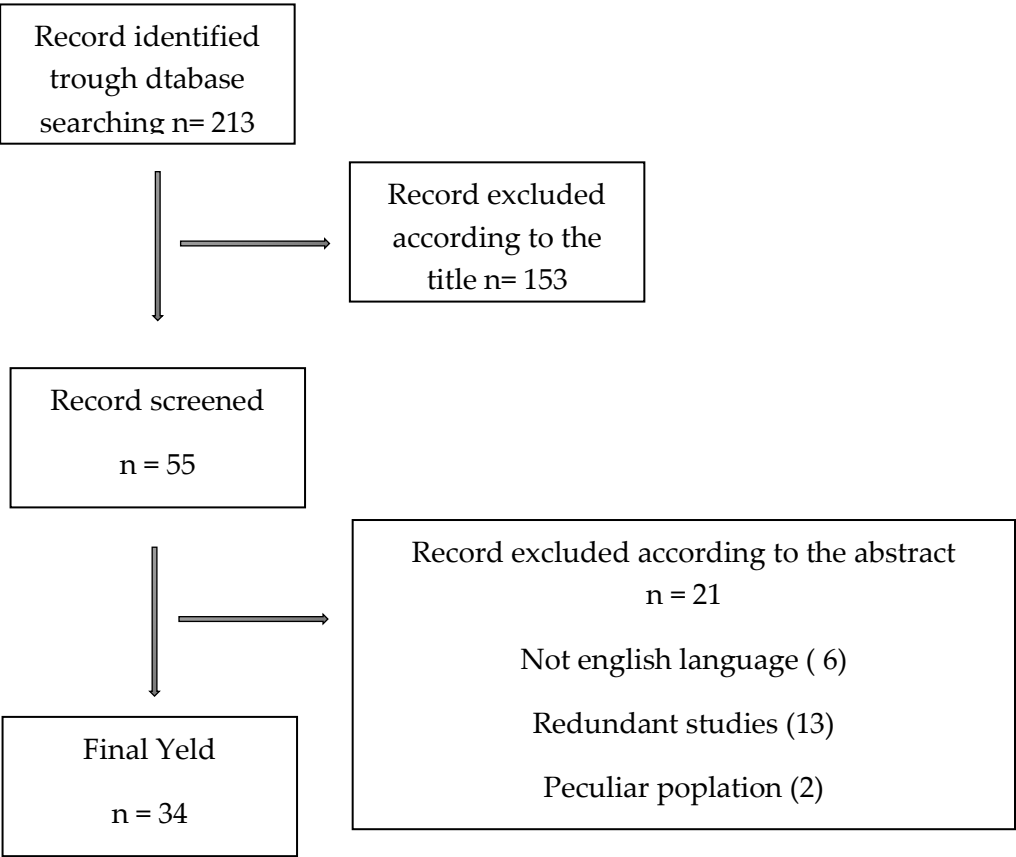

Supplement: Supplementary file 1 [file nutrients-13-04230-s001.zip › nutrients-1435356-supplementary.pdf]
